# Preoperative Decline and Postoperative Recovery of Wearable-Derived Physical Activity over a Four-Year Perioperative Period in Total Knee and Hip Arthroplasty

**DOI:** 10.3390/s26113319

**Published:** 2026-05-23

**Authors:** Yuezhou Zhang, Amos Folarin, Rongrong Zhong, Hyunju Kim, Callum Stewart, Shaoxiong Sun, Richard J. B. Dobson

**Affiliations:** 1 Department of Biostatistics & Health Informatics, Institute of Psychiatry, Psychology and Neuroscience, King’s College London, London SE5 8AF, UK; amos.folarin@kcl.ac.uk (A.F.); rongrong.zhong@kcl.ac.uk (R.Z.); hyunju.kim@kcl.ac.uk (H.K.); callum.stewart@kcl.ac.uk (C.S.); shaoxiong.sun@sheffield.ac.uk (S.S.); richard.j.dobson@kcl.ac.uk (R.J.B.D.); 2 Institute of Health Informatics, University College London, London NW1 2DA, UK; 3 NIHR Biomedical Research Centre, South London and Maudsley NHS Foundation Trust, London SE5 8AF, UK; 4 NIHR Biomedical Research Centre, University College London Hospitals NHS Foundation Trust, London NW1 2PG, UK; 5 Health Data Research UK, University College London, London NW1 2DA, UK; 6 Clinical Research Center & Division of Mood Disorders, Shanghai Mental Health Center, Shanghai Jiao Tong University School of Medicine, Shanghai 200030, China; 7 Department of Computer Science, University of Sheffield, Sheffield S1 4DP, UK

**Keywords:** total knee arthroplasty, total hip arthroplasty, wearable devices, physical activity, preoperative decline, postoperative recovery

## Abstract

**Highlights:**

**What are the main findings?**

- Wearable-derived step counts showed progressive preoperative decline and staged postoperative recovery over a four-year perioperative period in total knee and hip arthroplasty.
- Higher activity in the 4 weeks before arthroplasty was associated with a greater likelihood of recovery to longer-term preoperative habitual activity.

**What are the implications of the main findings?**

- Quantifying preoperative decline and staged postoperative recovery may help contextualize expectations for physical activity recovery after arthroplasty.
- Better preserved preoperative physical activity may be associated with subsequent step-count recovery, warranting validation in prospective studies.

**Abstract:**

We characterized long-term, objectively measured physical activity trajectories surrounding total knee arthroplasty (TKA) and total hip arthroplasty (THA) and examined factors associated with wearable-derived physical activity recovery. In this observational study within the All of Us Research Program, linked electronic health records and Fitbit step count data spanning the two years before and the two years after surgery were analyzed using piecewise linear mixed-effects models to characterize preoperative and postoperative trajectories. Recovery of physical activity was defined relative to two preoperative baselines—activity measured immediately before surgery and a more remote baseline approximating longer-term habitual activity—and associated factors were examined using Cox proportional hazards models. Among 238 participants (147 TKA, 91 THA; mean age 64.9 [SD 8.3] years), both procedures showed progressive preoperative decline, with accelerated decline beginning earlier in TKA than in THA. Postoperative recovery followed a staged pattern, with rapid early improvement, slower intermediate gains, and later stabilization. Recovery to the immediate preoperative baseline occurred earlier than recovery to the remote baseline. Higher activity during the 4 weeks before surgery was associated with a greater likelihood of recovery to the remote baseline. These findings support long-term wearable monitoring as a complementary measure of physical activity recovery after arthroplasty.

## 1. Introduction

Osteoarthritis is a prevalent and disabling condition associated with chronic pain, functional impairment, and reduced quality of life [1]. Total knee arthroplasty (TKA) and total hip arthroplasty (THA) are increasingly utilized worldwide and generally effective treatments for end-stage osteoarthritis [2]. However, a considerable proportion of patients report persistent pain [3,4], limited improvements in physical activity or functional performance [5,6,7], or dissatisfaction after surgery [8]. Emerging evidence further suggests that preoperative physical activity levels are associated with postoperative outcomes after TKA and THA [9,10]. Together, these findings underscore the need to characterize physical activity trajectories before and after arthroplasty and to identify factors associated with postoperative recovery.

Traditional assessments of preoperative status and postoperative recovery after TKA and THA rely largely on patient-reported outcome measures (PROMs) and intermittent clinical evaluations [11,12,13]. Although clinically informative, these approaches are inherently subjective, prone to recall bias, often limited by poor patient compliance, and unable to capture dynamic physical activity trajectories over time [14,15,16,17].

Wearable devices can complement traditional assessments by enabling cost-effective, objective monitoring of real-world physical activity over extended periods [18,19,20]. Devices such as Fitbit trackers can capture daily step count, a simple and intuitive measure of overall activity volume [21], with minimal user burden [22]. A growing number of studies have used wearable data to quantify postoperative recovery in physical activity after TKA and THA, demonstrating feasibility and providing objective benchmarks of physical activity recovery in real-world settings [23,24,25,26,27,28]. However, several important research gaps remain. First, preoperative decline in physical activity remains poorly characterized, in part because many wearable studies enroll participants only shortly before surgery, limiting the duration of preoperative monitoring and the ability to capture longer-term decline. Second, existing studies have largely focused on postoperative activity at prespecified time points (e.g., 1, 3, and 6 months) or descriptive trends rather than explicitly modeling recovery trajectories in physical activity. Third, recovery may be overestimated when it is defined relative to activity levels during a short period before surgery, as this period may already reflect disease-related decline and thus be lower than a person’s longer-term preoperative habitual activity level.

To address these gaps, longer-term, continuous wearable-derived physical activity data are needed across the perioperative period, particularly before surgery. The All of Us Research Program (AoURP) provides a unique resource linking longitudinal electronic health records (EHRs) with historical Fitbit-derived activity data, enabling retrospective characterization of physical activity trajectories over extended pre- and postoperative periods [29]. Therefore, this study aimed to characterize long-term, objectively measured physical activity trajectories surrounding TKA and THA, and to identify factors associated with postoperative wearable-derived physical activity recovery. Specifically, we used wearable-derived data from the two years before and two years after surgery to (1) quantify preoperative decline, (2) characterize staged postoperative recovery, and (3) examine how time to recovery and associated factors differ when recovery is defined relative to activity levels immediately before surgery versus a more remote preoperative baseline approximating longer-term habitual activity.

## 2. Materials and Methods

### 2.1. Data Source

The data used in this study were drawn from the AoURP controlled tier dataset (version 8; C2024Q3R8), including participants enrolled between May 2017 and October 2023. AoURP is an ongoing US National Institutes of Health-funded national longitudinal cohort, and its overall design and data collection procedures have been described previously [29,30]. In this data release version, EHR and Fitbit data were linked for 36,614 participants. For those who consented to share both data sources, historical (pre-enrollment) EHR records and Fitbit data were made available through participating healthcare provider organizations and linked Google Fitbit accounts, respectively [29,30,31]. All participants in the AoURP provided informed consent at enrollment, including consent for future secondary analyses of their data.

### 2.2. Ethical Statements

This study was conducted as a secondary analysis of de-identified data from the AoURP by authorized researchers within the secure Researcher Workbench platform. As the study did not involve direct interaction with human participants and used only existing de-identified data, it was exempt from additional institutional review board approval. All participants provided informed consent at the time of enrollment in the AoURP.

### 2.3. Identification of Arthroplasty Procedures

TKA and THA procedures and their dates were retrospectively identified from EHR data using Current Procedural Terminology (CPT-4) codes (TKA: CPT4-27447; THA: CPT4-27130). Eligible procedures were restricted to those occurring during periods with available Fitbit data. For participants with multiple eligible arthroplasties during the observation window, only the first procedure was included.

### 2.4. Comorbidity Assessment

Preoperative comorbidity burden was assessed using the Charlson Comorbidity Index (CCI) [32], derived from EHR diagnosis codes recorded before the arthroplasty date. ICD-9-CM and ICD-10-CM diagnosis codes were mapped to Charlson comorbidity categories using the widely used coding algorithm developed by Quan et al. [33]. A total CCI score was then calculated for each included participant. The corresponding diagnosis-code mapping table is provided in Supplementary Table S1.

### 2.5. Fitbit Step Count Data Processing and Perioperative Time Alignment

Daily step count data were obtained from linked Fitbit daily activity summary data. Step counts from the two years before and two years after surgery were aligned to the procedure date (day 0) and aggregated into consecutive, non-overlapping 7-day intervals to derive weekly averaged daily step counts. Preoperative time was indexed from weeks −104 to −1 and postoperative time from weeks 1 to 104, with postoperative week 1 corresponding to days 0–6. To ensure weekly data completeness, only weeks with at least 3 days of available daily step count records in the AoURP Fitbit daily activity summary data were retained.

### 2.6. Data Analysis

#### 2.6.1. Preoperative Trajectory Modeling

Preoperative physical activity trajectories were modeled separately for TKA and THA using piecewise linear mixed-effects models [34] with participant-specific random intercepts. The analysis covered weeks −104 to −1, with week −1 denoting the week immediately before surgery. Relative week was included as a continuous independent variable, with weekly averaged daily step count as the outcome. One knot (change point) was specified to estimate 2 preoperative phases, and the optimal knot was selected by grid search using the lowest Akaike information criterion (AIC). Models were adjusted for age, sex, body mass index (BMI), and CCI score, as these factors are established determinants of physical activity and may influence perioperative activity trajectories [35,36]. The mixed-effects models used all available weekly observations and did not require complete data at every time point, allowing participants with partially observed trajectories to contribute data for weeks with available Fitbit records. To balance data retention with trajectory reliability, participants were required to have at least 12 valid preoperative weeks of step count data, a threshold substantially higher than the minimum number of observations typically needed to estimate a longitudinal trajectory [37].

#### 2.6.2. Postoperative Trajectory Modeling

Based on prior literature [24,25,38,39] and visual inspection of the data, postoperative physical activity recovery was hypothesized to follow a staged trajectory. Accordingly, Fitbit step data during weeks 1–104 after surgery were modeled separately for TKA and THA using piecewise linear mixed-effects models with participant-specific random intercepts and 2 knots. Relative postoperative week was included as a continuous independent variable, with weekly averaged daily step count as the outcome. Optimal knot locations were identified by grid search using the lowest AIC. Similarly, models were adjusted for age, sex, BMI, and CCI score, and participants were required to have at least 12 valid postoperative weeks of step count data.

#### 2.6.3. Definitions of Wearable-Derived Physical Activity Recovery

Wearable-derived physical activity recovery was defined as the first of 2 consecutive weeks in which weekly averaged daily step count returned to or exceeded the prespecified preoperative baseline, in order to minimize misclassification due to short-term fluctuations.

We defined 2 preoperative physical activity baselines to evaluate recovery relative to each participant’s longer-term habitual activity level and short-term preoperative activity level: (1) the remote preoperative baseline, defined as the average daily step count during weeks −104 to −55, serving as an approximation of longer-term preoperative habitual activity; and (2) the immediate preoperative baseline, defined as the average daily step count during weeks −4 to −1, representing activity immediately preceding surgery.

#### 2.6.4. Time-to-Recovery Analysis

Time to wearable-derived physical activity recovery under each recovery definition was evaluated using Kaplan–Meier curves [40]. Cox proportional hazards (CoxPH) models [41] were used to examine associations between preoperative factors and time to recovery, reported as hazard ratios (HRs), where HR > 1 indicates a greater likelihood of recovery at any given time. Covariates included age, sex, marital status, employment status, education, income, BMI, CCI score, surgery type (TKA vs. THA), and the two preoperative baseline activity levels. Separate models were fitted for recovery defined using the remote and immediate preoperative baselines. The proportional hazards assumption was assessed using scaled Schoenfeld residuals [41]. Multicollinearity among covariates was assessed using the variance inflation factor (VIF) analysis, with VIF values below 5 interpreted as indicating no evidence of problematic multicollinearity [42]. To ensure calculable preoperative baselines and adequate postoperative follow-up, participants were required to have at least 1 valid week within the relevant baseline window and at least 12 valid postoperative weeks for inclusion in the time-to-recovery analysis.

#### 2.6.5. Sensitivity Analyses

Prespecified sensitivity analyses were conducted to assess the robustness of the main findings. First, to examine sensitivity to weekly Fitbit data completeness thresholds, we repeated the preoperative and postoperative piecewise linear mixed-effects trajectory analyses using a stricter inclusion criterion requiring available daily step count records for more than half of each weekly interval, defined as at least 4 days per week. Second, to evaluate the stability of the estimated knot locations and phase-specific slopes, we performed 200 participant-level bootstrap resampling iterations separately for the preoperative and postoperative trajectory analyses and summarized the distributions of the selected knots and corresponding slopes. Third, to assess sensitivity to the definition of physical activity recovery, we repeated the time-to-recovery analyses using a stricter recovery definition, requiring weekly averaged daily step count to reach or exceed the corresponding preoperative baseline for 4 consecutive weeks, rather than 2 consecutive weeks as in the primary analysis.

### 2.7. Data Availability

To ensure participant privacy, the data used in this study are available to approved researchers through the All of Us Research Workbench (https://workbench.researchallofus.org/login (accessed on 19 May 2026)) following registration, completion of required ethics training, and attestation of a data use agreement.

## 3. Results

### 3.1. Participant Characteristics

**Figure 1:**
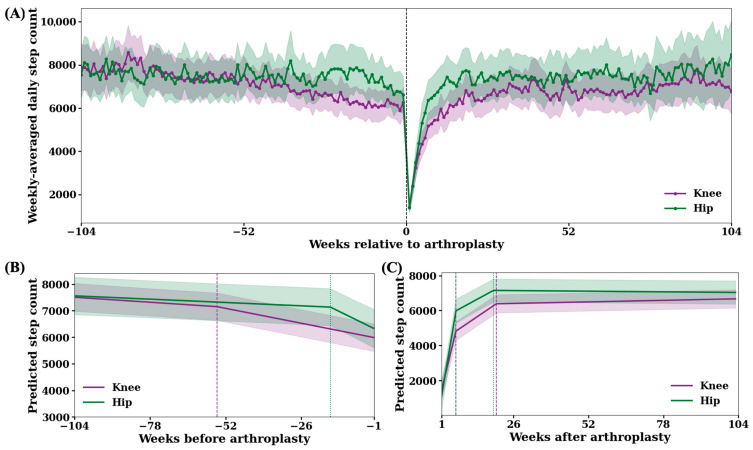
Observed and model-estimated perioperative physical activity trajectories in participants undergoing total knee arthroplasty (TKA) or total hip arthroplasty (THA). (**A**) Observed weekly averaged daily step counts from 104 weeks before to 104 weeks after arthroplasty. (**B**) Piecewise linear mixed-effects model–estimated preoperative decline trajectories from 104 weeks to 1 week before arthroplasty. (**C**) Piecewise linear mixed-effects model–estimated postoperative recovery trajectories from week 1 to week 104 after arthroplasty.

Sample sizes varied slightly across analytic cohorts because of differences in data availability and eligibility criteria for each analysis. Overall, 238 participants were included in at least 1 analytic cohort, including 147 TKA and 91 THA procedures. Mean age at consent was 64.9 (SD 8.3) years, 71.8% were female, and mean BMI was 30.9 (SD 6.6) kg/m^2^. Baseline sociodemographic and clinical characteristics are summarized in Table 1. Among participants included in the trajectory analyses, the median number of valid weeks was 83.5 (IQR 41.2–102.0) during the preoperative period and 78.0 (IQR 41.0–101.0) during the postoperative period; procedure-specific data completeness is summarized in Supplementary Table S2. Figure 1A shows observed weekly averaged daily step count trajectories for TKA and THA from 104 weeks before to 104 weeks after arthroplasty.

### 3.2. Preoperative Physical Activity Decline

Piecewise linear mixed-effects models identified distinct preoperative decline patterns in TKA and THA (Table 2; Figure 1B). At week −104, weekly averaged daily step counts were similar between groups (TKA 7506.4 [95% CI 6978.4–8034.4] vs. THA 7561.1 [6853.8–8268.4]). During weeks −104 to −55 in TKA and weeks −104 to −16 in THA, both groups showed only modest declines (TKA −7.2 and THA −4.8 steps/week; both *p* < 0.001). Thereafter, trajectories diverged: decline accelerated earlier in TKA, beginning at week −55 (−21.6 steps/week; *p* < 0.001), whereas THA remained gradual until week −16, followed by a steeper decline in the final preoperative months (−53.9 steps/week; *p* < 0.001). By week −1, weekly averaged daily step counts had decreased to 5986.9 [5466.0–6507.8] in TKA and 6328.2 [5602.8–7053.5] in THA.

### 3.3. Postoperative Physical Activity Recovery

Three-phase piecewise linear mixed-effects models identified staged postoperative recovery in both procedures (Table 3; Figure 1C), with optimal knots at weeks 6 and 20 in TKA and weeks 6 and 19 in THA. During weeks 1–6, weekly averaged daily step counts increased rapidly in both groups, with a steeper slope in THA than in TKA (+970.1 vs. +668.4 steps/week; both *p* < 0.001). Improvement then slowed during weeks 7–20 in TKA and 7–19 in THA (TKA +111.6 and THA +89.9 steps/week; both *p* < 0.001). Thereafter, trajectories plateaued through week 104, with minimal weekly changes (TKA +3.4; THA −1.4 steps/week), reaching estimated weekly averaged daily step counts of 6671.1 [6145.3–7196.9] in TKA and 7035.9 [6365.6–7706.2] in THA at week 104.

### 3.4. Wearable-Derived Physical Activity Recovery Under Different Baseline Definitions

**Figure 2:**
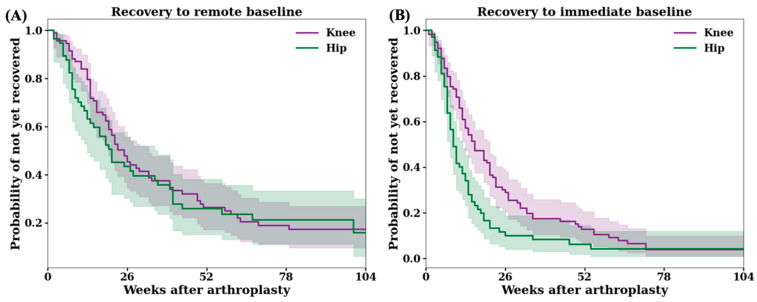
Kaplan–Meier curves for time to physical activity recovery following total knee arthroplasty (TKA) and total hip arthroplasty (THA) under 2 preoperative baseline definitions. (**A**) Recovery to the remote preoperative baseline, defined as the mean daily step count during weeks −104 to −55 before arthroplasty and approximating longer-term preoperative habitual activity. (**B**) Recovery to the immediate preoperative baseline, defined as the mean daily step count during weeks −4 to −1 before arthroplasty and representing short-term preoperative activity.

Kaplan–Meier curves for recovery under the 2 baseline definitions are shown in Figure 2. Using the remote preoperative baseline, 152 participants were eligible (TKA 95; THA 57), and 112 (73.7%) recovered within 104 weeks after arthroplasty. Median time to recovery was 22 (95% CI 20–29) weeks, with no significant difference between procedures. Using the immediate preoperative baseline, 185 participants were eligible, and 158 (85.4%) recovered within 104 weeks. Median time to recovery was 13 (95% CI 11–15) weeks and was shorter in THA than in TKA (9 [95% CI 8–12] vs. 16 [95% CI 13–21] weeks; log-rank *p* = 0.001). Procedure-specific counts of non-recovery or censoring are not reported because AoURP policy prohibits reporting cell sizes smaller than 20.

### 3.5. Factors Associated with Time to Recovery

In the CoxPH models, recovery to the remote preoperative physical activity baseline was more likely in participants with higher immediate preoperative activity level (HR 1.47 per 1000 steps; *p* < 0.001) and less likely in those with higher remote baseline activity level (HR 0.69 per 1000 steps; *p* < 0.001) or older age (HR 0.96 per year; *p* = 0.01) (Table 4). For recovery to the immediate preoperative baseline, surgery type was the only significant factor, with lower likelihood of recovery after TKA than THA (HR 0.59; *p* = 0.001; Table 5). VIF analysis showed no evidence of problematic multicollinearity among model covariates (Supplementary Table S5).

### 3.6. Sensitivity Analyses

In sensitivity analyses requiring at least 4 days of available daily step count records per week, the main findings were largely unchanged. Compared with the primary analysis, the number of participants included in the preoperative and postoperative trajectory models decreased modestly by 13 and 4 participants, respectively. The selected preoperative knot shifted slightly from week −55 to week −53 for TKA and remained unchanged at week −16 for THA. Postoperative knots were also similar, occurring at weeks 6 and 19 for both TKA and THA. The estimated slopes and time-to-recovery results were broadly consistent with the primary analyses, supporting the robustness of the findings to a stricter weekly data completeness threshold. Detailed coefficient estimates from the piecewise linear mixed-effects models and CoxPH model results are provided in Supplementary Tables S6–S9.

Bootstrap analyses further supported the stability of the selected knots and corresponding phase-specific slopes. In the preoperative models, the median selected knots were identical to the primary estimates for both TKA and THA, occurring at week −55 for TKA (IQR −57 to −53) and week −16 for THA (IQR −18 to −14). In the postoperative models, the median first knot was week 6 for both procedures (TKA IQR 5 to 7; THA IQR 6 to 7), and the median second knot was week 19 for both procedures (TKA IQR 19 to 21; THA IQR 18 to 20). Phase-specific slope estimates were also broadly consistent with the primary analyses. Detailed bootstrap results are provided in Supplementary Tables S10 and S11.

Using a stricter recovery definition requiring 4 consecutive weeks at or above the corresponding preoperative baseline reduced the number of participants classified as recovered, as expected, with 84 (55.3%) recovering to the remote baseline and 135 (73.0%) recovering to the immediate baseline. Nevertheless, the CoxPH model findings were consistent with the primary analyses (Supplementary Tables S12 and S13).

## 4. Discussion

In this retrospective analysis of adults undergoing TKA or THA within the AoURP, linked EHR and wearable step count data enabled characterization of perioperative physical activity trajectories over a four-year period. Three main findings emerged. First, physical activity declined progressively before surgery, highlighting a preoperative phase that has been insufficiently characterized in prior wearable studies. Second, postoperative physical activity recovery followed a staged pattern, with rapid early improvement, slower intermediate gains, and subsequent stabilization. Third, recovery rates and time to recovery differed according to whether recovery was defined relative to immediate preoperative activity levels or a more remote preoperative baseline approximating longer-term preoperative habitual activity. In addition, participants with higher activity levels immediately before surgery were more likely to recover to longer-term habitual preoperative activity levels. Together, these findings highlight the potential of long-term wearable monitoring to complement perioperative assessment by providing objective information on physical activity trajectories after arthroplasty.

Our analyses extend prior work by quantifying long-term preoperative physical activity trajectories, a phase that has been insufficiently captured in prior wearable studies. Both TKA and THA showed progressive preoperative decline, although accelerated decline began earlier in TKA than in THA. This may reflect differences in joint biomechanics [43], disease course, or timing of surgery, consistent with prior work suggesting that THA may occur earlier in the osteoarthritis course than TKA [44]. However, these procedure-specific patterns should be interpreted cautiously and require further validation.

Postoperative physical activity recovery in both procedures followed a staged pattern, with rapid early improvement, slower intermediate gains, and later stabilization. This pattern is broadly consistent with prior studies reporting early postoperative gains and later plateauing in physical activity after arthroplasty [24,25,38,39], but extends existing work by showing that recovery can be characterized as a staged trajectory rather than assessed only at isolated postoperative time points. Notably, the early recovery slope was steeper in THA than in TKA, consistent with previous reports of faster early functional improvement following hip arthroplasty [23,45]. Given the observational design, missingness in longitudinal wearable data, and potential selection bias, the estimated knot locations should be interpreted as approximate transition points rather than definitive clinical cut-points. Thus, these findings should be viewed as evidence of distinct wearable-derived perioperative activity trajectory phases in real-world settings, while the exact timing and slopes of these phases may vary across populations.

An important methodological insight from this study is that postoperative physical activity recovery depended on how the preoperative baseline was defined. In prior wearable studies, limited preoperative monitoring often meant that recovery was defined relative to activity levels measured only shortly before surgery [23,26,46]. Our findings suggest that this approach may overestimate recovery, because activity levels immediately before surgery may already reflect disease-related decline [47] rather than typical functional capacity. Consistent with this interpretation, recovery to the immediate preoperative baseline occurred substantially earlier than recovery to the remote baseline, with a median difference of approximately 9 weeks. A more remote preoperative baseline may therefore provide a more informative benchmark for interpreting recovery relative to immediate preoperative activity, although it should not be interpreted as a definitive measure of pre-disease habitual activity. Nevertheless, because wearable-derived physical activity recovery after arthroplasty does not yet have a well-established definition, our time-to-recovery analyses should be interpreted as exploratory rather than as establishing a definitive threshold for stable functional recovery.

Our survival analyses further suggested that participants with higher activity levels immediately before surgery were more likely to recover to their longer-term preoperative habitual activity level, indicating an association between better preserved preoperative physical activity and more favorable step-count recovery. This finding is consistent with prior evidence linking preoperative functional status to postoperative outcomes [9,10,48]. Older age was also associated with a lower likelihood of recovery, in line with previous longitudinal studies of arthroplasty outcomes [49,50,51]. As expected, higher remote baseline activity was associated with a lower likelihood of recovery within follow-up, likely reflecting a higher recovery threshold. These associations should not be interpreted as causal, and the underlying mechanisms remain unclear. Although we adjusted for age, sex, BMI, and CCI, residual confounding by pain severity, joint-specific function, motivation, access to rehabilitation, and other clinical or behavioral factors may remain. These findings suggest that preoperative activity status may help contextualize expectations for physical activity recovery, although its role in broader perioperative decision-making requires validation alongside pain, function, patient-reported outcomes, and prospectively defined clinical thresholds.

This study was not designed to develop clinical prediction tools or intervention thresholds. Instead, the findings should be viewed as hypothesis-generating evidence that long-term wearable data can provide complementary information on physical activity trajectories; prospective validation is needed before clinical implementation.

This study has several limitations. First, the bring-your-own-device design of wearable data collection in AoURP may introduce selection bias toward individuals who are more health-conscious or more engaged with digital technology. Therefore, the observed trajectories may not fully generalize to patients without wearable devices or to populations with lower digital access or engagement. Second, missingness is an inherent limitation of long-term passively collected wearable data. We defined data availability using daily records in the AoURP Fitbit daily activity summary data rather than an hourly step-count-based wear-time rule [52], because such a rule could misclassify true low-activity days during early postoperative recovery as non-wear. Although sensitivity analyses supported the robustness of the trajectory findings, incomplete data may still have influenced the estimated trajectories, and future prospective studies with standardized device deployment and wear-time assessment are needed to validate these findings.

Third, although the retrospective design enabled extended preoperative observation, complementary clinical measures such as pain severity, joint-specific function, performance-based mobility tests, and PROMs were not consistently available. Therefore, step-count recovery should be interpreted as one objective dimension of postoperative recovery rather than a comprehensive measure of multidimensional clinical recovery. The observed association between preoperative activity and step-count recovery may also be influenced by residual confounding, and this study was not designed to determine the causal mechanisms underlying this association. Fourth, although we used a remote preoperative baseline to approximate longer-term preoperative habitual activity, osteoarthritis-related functional decline may begin several years before surgery [53]. Therefore, this baseline may still reflect some degree of disease-related decline rather than true pre-disease activity, and longer-duration prospective wearable data are needed to capture earlier stages of decline and validate recovery estimates based on the remote preoperative baseline. Finally, despite the scale of AoURP, the subset of participants with longitudinal wearable data and confirmed arthroplasty remained relatively modest, limiting precision and warranting future validation in larger and more diverse cohorts, particularly for procedure-specific differences between TKA and THA.

## 5. Conclusions

Using long-term wearable data linked to EHRs, this study helps address an important gap in objective characterization of perioperative physical activity surrounding arthroplasty, particularly before surgery. Physical activity showed progressive preoperative decline and staged postoperative recovery, while physical activity recovery estimates differed according to whether recovery was defined relative to immediate or remote preoperative activity levels. These findings suggest that preoperative activity status and baseline definition are important for interpreting wearable-derived physical activity recovery and support the potential of long-term wearable monitoring to complement postoperative assessment.

## Figures and Tables

**Table 1:** Sociodemographic and clinical characteristics of included participants undergoing total knee arthroplasty (TKA) or total hip arthroplasty (THA) in the All of Us Research Program.

Characteristic	Overall (*n* = 238)	THA (*n* = 91)	TKA (*n* = 147)	*p* Value
Age, mean (SD)	64.9 (8.3)	64.0 (9.0)	65.5 (7.8)	0.19
BMI, mean (SD)	30.9 (6.6)	29.9 (6.4)	31.5 (6.7)	0.07
Sex, n (%)				0.25
Female	171 (71.8)	61 (67.0)	110 (74.8)	
Male	67 (28.2)	30 (33.0)	37 (25.2)	
Charlson Comorbidity Index, mean (SD)	2.1 (2.5)	1.8 (2.3)	2.4 (2.6)	0.07
Education, n (%)				0.35
College degree	170 (71.4)	67 (73.6)	103 (70.1)	
Some college/no college	68 (28.6)	24 (26.4)	44 (29.9)	
Income, n (%)				0.06
High	50 (21.0)	26 (28.6)	24 (16.3)	
Mid/Low/Skip	188 (79.0)	65 (71.4)	123 (83.7)	
Employment, n (%)				0.18
Working	88 (37.0)	39 (42.9)	49 (33.3)	
Not working	150 (63.0)	52 (57.1)	98 (66.7)	
Marital status, n (%)				>0.99
Married/Living with partner	159 (66.8)	61 (67.0)	98 (66.7)	
Never married/Divorced/Widowed/Separated	79 (33.2)	30 (33.0)	49 (33.3)	

BMI, body mass index. Education was categorized into three groups: college degree (college graduate or advanced degree), some college (one to three years of college), and no college (high school/GED or less). Income was grouped into three levels based on reported household income: high (≥$150,000), mid ($50,000–$150,000), and low/skip (<$50,000, prefer not to answer, or skipped). Employment was categorized as working (employed for wages or self-employed) and not working (retired, homemaker, unable to work, or unemployed). Marital status was categorized as married/living with partner and never married/divorced/widowed/separated. In accordance with All of Us Research Program reporting policies, categories with fewer than 20 participants in any cell cannot be reported to protect participant confidentiality; therefore, some categories were combined, and race and ethnicity information are not displayed.

**Table 2:** Estimated weekly changes in daily step count during the preoperative period in participants undergoing total knee arthroplasty (TKA) or total hip arthroplasty (THA), based on piecewise linear mixed-effects models.

Group	Phase	Weekly Change in Daily Step Count, β (Steps/Week)	95% CI	*p* Value
TKA (*n* = 115)	Gradual decline phase (weeks −104 to −55)	−7.2	−11.0 to −3.4	<0.001
	Accelerated decline phase (weeks −54 to −1)	−21.6	−24.7 to −18.5	<0.001
THA (*n* = 75)	Gradual decline phase (weeks −104 to −16)	−4.8	−7.0 to −2.6	<0.001
	Accelerated decline phase (weeks −15 to −1)	−53.9	−70.7 to −37.0	<0.001

Time was indexed relative to the procedure date and spanned the preoperative period from week −104 to week −1. Slopes (β) were estimated using two-phase piecewise linear mixed-effects models with participant-specific random intercepts. Knot locations were selected using the Akaike Information Criterion (AIC). Full fixed-effect estimates from the preoperative piecewise linear mixed-effects models are provided in Supplementary Table S3.

**Table 3:** Estimated weekly changes in daily step count during the postoperative period in participants undergoing total knee arthroplasty (TKA) or total hip arthroplasty (THA), based on three-phase piecewise linear mixed-effects models.

Group	Phase	Weekly Change in Daily Step Count, β (Steps/Week)	95% CI	*p* Value
TKA (*n* = 120)	Rapid recovery phase (weeks 1–6)	668.4	593.1 to 743.7	<0.001
	Decelerating recovery phase (weeks 7–20)	111.6	96.9 to 126.4	<0.001
	Plateau phase (weeks 21–104)	3.4	1.4 to 5.4	0.001
THA (*n* = 81)	Rapid recovery phase (weeks 1–6)	970.1	880.3 to 1059.9	<0.001
	Decelerating recovery phase (weeks 7–19)	89.9	70.8 to 108.8	<0.001
	Plateau phase (weeks 20–104)	−1.4	−3.8 to 1.1	0.27

Time was indexed relative to the procedure date and spanned the postoperative period from week 1 to week 104 (week 1 corresponds to days 0–6 postoperatively). Slopes (β) were estimated using three-phase piecewise linear mixed-effects models with participant-specific random intercepts. Knot locations were selected using an Akaike Information Criterion (AIC)-based grid search. Full fixed-effect estimates from the postoperative piecewise linear mixed-effects models are provided in Supplementary Table S4.

**Table 4:** Multivariable Cox proportional hazards model for time to recovery to the remote preoperative physical activity baseline, defined as the mean daily step count during weeks −104 to −55 before surgery and approximating longer-term preoperative habitual activity.

Predictor	Hazard Ratio (HR)	95% CI	*p* Value
Age (per year)	0.96	0.94–0.99	0.01
BMI (per kg/m^2^)	0.99	0.96–1.02	0.52
Male sex (reference: female)	1.14	0.77–1.70	0.52
Charlson Comorbidity Index, per point	0.96	0.89–1.05	0.36
Surgery type: TKA (reference: THA)	0.96	0.62–1.49	0.86
Education: no college (reference: college)	1.24	0.58–2.64	0.57
Education: some college (reference: college)	1.20	0.76–1.88	0.43
Employment: working (reference: not working)	0.66	0.41–1.04	0.08
Income: low/skip (reference: high)	1.14	0.66–1.96	0.64
Income: mid (reference: high)	0.73	0.45–1.20	0.21
Marital status: not married (reference: married)	0.86	0.56–1.32	0.48
Immediate preoperative activity level (per 1000 steps)	1.47	1.28–1.67	<0.001
Remote preoperative activity level (per 1000 steps)	0.69	0.59–0.79	<0.001

Immediate preoperative activity level was defined as the mean daily step count during weeks −4 to −1 before surgery. Remote preoperative activity level was defined as the mean daily step count during weeks −104 to −55 before surgery. HRs greater than 1 indicate a higher likelihood of achieving recovery in physical activity. The proportional hazards assumption was not violated. Definitions of categorical variables are detailed in the footnote of Table 1.

**Table 5:** Multivariable Cox proportional hazards model for time to recovery to the immediate preoperative physical activity baseline, defined as the mean daily step count during weeks −4 to −1 before surgery and representing short-term preoperative activity.

Predictor	Hazard Ratio (HR)	95% CI	*p* Value
Age (per year)	0.98	0.95–1.01	0.15
BMI (per kg/m^2^)	1.01	0.96–1.04	0.98
Male sex (reference: female)	0.75	0.46–1.21	0.24
Charlson Comorbidity Index, per point	0.99	0.87–1.12	0.83
Surgery type: TKA (reference: THA)	0.59	0.38–0.90	0.001
Education: no college (reference: college)	1.03	0.67–1.60	0.89
Education: some college (reference: college)	1.04	0.62–1.76	0.87
Employment: working (reference: not working)	0.87	0.55–1.38	0.56
Income: low/skip (reference: high)	1.52	0.87–2.63	0.14
Income: mid (reference: high)	1.41	0.87–2.29	0.17
Marital status: not married (reference: married)	0.68	0.44–1.06	0.09
Immediate preoperative activity level (per 1000 steps)	0.90	0.80–1.01	0.07
Remote preoperative activity level (per 1000 steps)	1.08	0.97–1.20	0.18

Immediate preoperative activity level was defined as the mean daily step count during weeks −4 to −1 before surgery. Remote preoperative activity level was defined as the mean daily step count during weeks −104 to −55 prior to surgery. HRs greater than 1 indicate a higher likelihood of achieving recovery in physical activity. The proportional hazards assumption was assessed using scaled Schoenfeld residuals and was not violated. Definitions of categorical variables are detailed in the footnote of Table 1.

## Data Availability

To ensure participant privacy, the data used in this study are available to approved researchers through the All of Us Research Workbench (https://workbench.researchallofus.org/login (19 May 2026)) following registration, completion of required ethics training, and attestation of a data use agreement. Public release of individual-level participant data is not permitted under the All of Us Research Program privacy policies.

## Supplementary Materials

The following supporting information can be downloaded at: https://www.mdpi.com/article/10.3390/s26113319/s1, Table S1: ICD-9-CM and ICD-10-CM Diagnosis Code Mapping for Charlson Comorbidity Index Categories; Table S2: Fitbit data completeness in the preoperative and postoperative trajectory analysis cohorts; Table S3: Fixed-effect estimates from preoperative piecewise linear mixed-effects models for weekly averaged daily step count before arthroplasty; Table S4: Fixed-effect estimates from postoperative piecewise linear mixed-effects models for weekly averaged daily step count after arthroplasty; Table S5: Variance inflation factor (VIF) analysis for covariates in the Cox proportional hazards models; Table S6: Preoperative piecewise linear mixed-effects models requiring at least 4 days of available Fitbit step count data per week; Table S7: Postoperative piecewise linear mixed-effects models requiring at least 4 days of available Fitbit step count data per week; Table S8: Cox proportional hazards model for recovery to the remote preoperative baseline requiring at least 4 days of available Fitbit step count data per week; Table S9: Cox proportional hazards model for recovery to the immediate preoperative baseline requiring at least 4 days of available Fitbit step count data per week; Table S10: Bootstrap stability analysis of preoperative knots and phase-specific slopes; Table S11: Bootstrap stability analysis of postoperative knots and phase-specific slopes; Table S12: Cox proportional hazards model for recovery to the remote preoperative baseline, defined as 4 consecutive weeks at or above baseline; Table S13: Cox proportional hazards model for recovery to the immediate preoperative baseline, defined as 4 consecutive weeks at or above baseline.
