# Supplementary material for: Preoperative Decline and Postoperative Recovery of Wearable-Derived Physical Activity over a Four-Year Perioperative Period in Total Knee and Hip Arthroplasty
Source: Sensors (Basel). 2026 May 23;26(11):3319. doi: 10.3390/s26113319 (PMC13259192; doi:10.3390/s26113319)
Supplement: Supplementary file 1 [file sensors-26-03319-s001.zip › sensors-4302566-supplementary.pdf]

Table S1. ICD-9-CM and ICD-10-CM Diagnosis Code Mapping for Charlson Comorbidity Index Categories

Comorbidities	Score	ICD-9-CM	ICD-10-CM
Myocardial infarction	1	410.x, 412.x	I21.x, I22.x, I25.2
Congestive heart failure	1	398.91, 402.01, 402.11, 402.91, 404.01, 404.03, 404.11, 404.13, 404.91, 404.93, 425.4–425.9, 428.x	I09.9, I11.0, I13.0, I13.2, I25.5, I42.0, I42.5–I42.9, I43.x, I50.x, P29.0
Peripheral vascular disease	1	093.0, 437.3, 440.x, 441.x, 443.1–443.9, 447.1, 557.1, 557.9, V43.4	I70.x, I71.x, I73.1, I73.8, I73.9, I77.1, I79.0, I79.2, K55.1, K55.8, K55.9, Z95.8, Z95.9
Cerebrovascular disease	1	362.34, 430.x–438.x	G45.x, G46.x, H34.0, I60.x–I69.x
Dementia	1	290.x, 294.1, 331.2	F00.x–F03.x, F05.1, G30.x, G31.1
Chronic pulmonary disease	1	416.8, 416.9, 490.x–505.x, 506.4, 508.1, 508.8	I27.8, I27.9, J40.x–J47.x, J60.x–J67.x, J68.4, J70.1, J70.3
Rheumatologic disease	1	446.5, 710.0–710.4, 714.0–714.2, 714.8, 725.x	M05.x, M06.x, M31.5, M32.x–M34.x, M35.1, M35.3, M36.0
Peptic ulcer disease	1	531.x–534.x	K25.x–K28.x
Mild liver disease	1	070.22, 070.23, 070.32, 070.33, 070.44, 070.54, 070.6, 070.9, 570.x, 571.x, 573.3, 573.4, 573.8, 573.9, V42.7	B18.x, K70.0–K70.3, K70.9, K71.3–K71.5, K71.7, K73.x, K74.x, K76.0, K76.2–K76.4, K76.8, K76.9, Z94.4
Diabetes without chronic complication	1	250.0–250.3, 250.8, 250.9	E10.0, E10.1, E10.6, E10.8, E10.9, E11.0, E11.1, E11.6, E11.8, E11.9, E12.0, E12.1, E12.6, E12.8, E12.9, E13.0, E13.1, E13.6, E13.8, E13.9, E14.0, E14.1, E14.6, E14.8, E14.9
Diabetes with chronic complication	2	250.4–250.7	E10.2–E10.5, E10.7, E11.2–E11.5, E11.7, E12.2–E12.5, E12.7, E13.2–E13.5, E13.7, E14.2–E14.5, E14.7
Hemiplegia or paraplegia	2	334.1, 342.x, 343.x, 344.0–344.6, 344.9	G04.1, G11.4, G80.1, G80.2, G81.x, G82.x, G83.0–G83.4, G83.9
Renal disease	2	403.01, 403.11, 403.91, 404.02, 404.03, 404.12, 404.13, 404.92, 404.93, 582.x, 583.0–583.7, 585.x, 586.x, 588.0, V42.0, V45.1, V56.x	I12.0, I13.1, N03.2–N03.7, N05.2–N05.7, N18.x, N19.x, N25.0, Z49.0–Z49.2, Z94.0, Z99.2
Any malignancy, including leukemia and lymphoma, except malignant neoplasm of skin	2	140.x–172.x, 174.x–195.x, 200.x–208.x, 238.6	C00.x–C26.x, C30.x–C34.x, C37.x–C41.x, C43.x, C45.x–C58.x, C60.x–C76.x, C81.x–C85.x, C88.x, C90.x–C97.x
Moderate or severe liver disease	3	456.0–456.2, 572.2–572.8	I85.0, I85.9, I86.4, I98.2, K70.4, K71.1, K72.1, K72.9, K76.5–K76.7
Metastatic solid tumor	6	196.x–199.x	C77.x–C80.x
AIDS / HIV	6	042.x–044.x	B20.x–B22.x, B24.x

Table S2. Fitbit data completeness in the preoperative and postoperative trajectory analysis cohorts

Analysis cohort	Procedure	Participants, n	Valid weeks, median	Valid weeks, IQR
Preoperative	TKA	115	85	44.0–102.0
	THA	75	83	36.0–102.0
Postoperative	TKA	120	77	40.8–100.0
	THA	81	78	41.0–102.0

Note. TKA, total knee arthroplasty; THA, total hip arthroplasty; IQR, interquartile range. Valid weeks were defined as weeks with at least 3 days of available daily step count records in the AoURP Fitbit daily activity summary data. The preoperative analysis window spanned weeks −104 to −1, and the postoperative analysis window spanned weeks 1 to 104 relative to the arthroplasty date.

Table S3. Fixed-effect estimates from preoperative piecewise linear mixed-effects models for weekly averaged daily step count before arthroplasty.

Procedure	Predictor	β	95% CI	P value
TKA	Intercept	18,042.50	11,715.4 to 24,369.6	<0.001
	Male sex	1,933.30	733.5 to 3,133.1	0.002
	Gradual decline phase, weeks −104 to −55	−7.2	−11.0 to −3.4	<0.001
	Accelerated decline phase, weeks −54 to −1	−21.6	−24.7 to −18.5	<0.001
	Age at consent, per year	−75.4	−147.3 to −3.5	0.04
	BMI, per kg/m²	−188.5	−274.1 to −102.9	<0.001
	Charlson Comorbidity Index, per point	−422.8	−642.4 to −203.3	<0.001
THA	Intercept	12,885.30	6,501.2 to 19,269.3	<0.001
	Male sex	902.1	−628.7 to 2,433.0	0.25
	Gradual decline phase, weeks −104 to −16	−4.8	−7.0 to −2.6	<0.001
	Accelerated decline phase, weeks −15 to −1	−53.9	−70.7 to −37.0	<0.001
	Age at consent, per year	32.9	−45.2 to 110.9	0.41
	BMI, per kg/m²	−252.5	−363.3 to −141.6	<0.001
	Charlson Comorbidity Index, per point	−383.4	−715.3 to −51.5	0.02

Note. Time was indexed relative to the procedure date and spanned the preoperative period from week −104 to week −1. Slopes (β) were estimated using two-phase piecewise linear mixed-effects models with participant-specific random intercepts. Knot locations were selected using the Akaike Information Criterion (AIC).

Table S4. Fixed-effect estimates from postoperative piecewise linear mixed-effects models for weekly averaged daily step count after arthroplasty.

Procedure	Predictor	β	95% CI	P value
TKA	Intercept	13,043.60	6,201.3 to 19,885.9	<0.001
	Male sex	1,742.90	540.5 to 2,945.2	0.004
	Rapid recovery phase, weeks 1–6	668.4	593.1 to 743.7	<0.001
	Decelerating recovery phase, weeks 7–20	111.6	96.9 to 126.4	<0.001
	Plateau phase, weeks 21–104	3.4	1.4 to 5.4	0.001
	Age at consent, per year	−96.3	−172.2 to −20.4	0.01
	BMI, per kg/m²	−179.4	−270.3 to −88.4	<0.001
	Charlson Comorbidity Index, per point	−328.5	−522.5 to −134.5	0.001
THA	Intercept	7,613.10	1,264.5 to 13,961.7	0.02
	Male sex	1,300.00	−150.5 to 2,750.5	0.08
	Rapid recovery phase, weeks 1–6	970.1	880.3 to 1,059.9	<0.001
	Decelerating recovery phase, weeks 7–19	89.9	70.8 to 108.8	<0.001
	Plateau phase, weeks 20–104	−1.4	−3.8 to 1.1	0.27
	Age at consent, per year	2.6	−73.8 to 79.0	0.95
	BMI, per kg/m²	−248.9	−354.6 to −143.2	<0.001
	Charlson Comorbidity Index, per point	−416.9	−707.1 to −126.8	0.005

Note. Time was indexed relative to the procedure date and spanned the postoperative period from week 1 to week 104 (week 1 corresponds to days 0–6 postoperatively). Slopes (β) were estimated using three-phase piecewise linear mixed-effects models with participant-specific random intercepts. Knot locations were selected using an Akaike Information Criterion (AIC)–based grid search.

Table S5. Variance inflation factor (VIF) analysis for covariates in the Cox proportional hazards models

**Predictor**	**VIF**
Age (per year)	1.56
BMI (per kg/m^2)	1.66
Male sex (reference: female)	1.11
Charlson Comorbidity Index, per point	1.26
Surgery type: TKA (reference: THA)	1.1
Education: no college (reference: college)	1.14
Education: some college (reference: college)	1.24
Employment: working (reference: not working)	1.36
Income: low/skip (reference: high)	1.82
Income: mid (reference: high)	1.71
Marital status: not married (reference: married)	1.19
Immediate preoperative activity level (per 1000 steps)	3.58
Remote preoperative activity level (per 1000 steps)	3.63

Note. VIF values were calculated for covariates included in the Cox proportional hazards models. All VIF values were below 5, indicating no evidence of problematic multicollinearity among model covariates, including immediate and remote preoperative activity levels.

Table S6. Preoperative piecewise linear mixed-effects models requiring at least 4 days of available Fitbit step count data per week.

Procedure	Predictor	β	95% CI	P value
TKA	Intercept	18,055.90	11,725.5 to 24,386.2	<0.001
	Male sex	1,938.20	737.6 to 3,138.8	0.002
	Gradual decline phase, weeks −104 to −53	−7.4	−11.1 to −3.8	<0.001
	Accelerated decline phase, weeks −52 to −1	−22.2	−25.4 to −18.9	<0.001
	Age at consent, per year	−75.6	−147.6 to −3.6	0.04
	BMI, per kg/m²	−188.2	−273.9 to −102.6	<0.001
	Charlson Comorbidity Index, per point	−425.2	−644.9 to −205.5	<0.001
THA	Intercept	12,129.60	5,657.4 to 18,601.8	<0.001
	Male sex	1,119.70	−431.3 to 2,670.7	0.157
	Gradual decline phase, weeks −104 to −16	−4.5	−6.7 to −2.3	<0.001
	Accelerated decline phase, weeks −15 to −1	−57.2	−74.1 to −40.3	<0.001
	Age at consent, per year	42.6	−36.4 to 121.5	0.291
	BMI, per kg/m²	−244.1	−355.1 to −133.1	<0.001
	Charlson Comorbidity Index, per point	−412.2	−744.0 to −80.3	0.015

Table S7. Postoperative piecewise linear mixed-effects models requiring at least 4 days of available Fitbit step count data per week.

Procedure	Predictor	β	95% CI	P value
TKA	Intercept	13,488.30	6,596.8 to 20,379.7	<0.001
	Male sex	1,758.90	536.9 to 2,981.0	0.005
	Rapid recovery phase, weeks 1–6	656.9	578.8 to 735.0	<0.001
	Decelerating recovery phase, weeks 7–19	123	106.7 to 139.2	<0.001
	Plateau phase, weeks 20–104	3.8	1.7 to 5.8	<0.001
	Age at consent, per year	−99.4	−175.4 to −23.4	0.01
	BMI, per kg/m^2	−183.5	−275.7 to −91.4	<0.001
	Charlson Comorbidity Index, per point	−342.0	−536.7 to −147.3	0.001
THA	Intercept	7,569.40	1,192.2 to 13,946.5	0.02
	Male sex	1,427.90	−50.3 to 2,906.2	0.058
	Rapid recovery phase, weeks 1–6	973.2	882.3 to 1,064.0	<0.001
	Decelerating recovery phase, weeks 7–19	88.5	69.4 to 107.5	<0.001
	Plateau phase, weeks 20–104	−1.0	−3.5 to 1.4	0.402
	Age at consent, per year	2.9	−73.8 to 79.7	0.94
	BMI, per kg/m^2	−249.1	−355.2 to −143.0	<0.001
	Charlson Comorbidity Index, per point	−401.4	−694.7 to −108.1	0.007

Table S8. Cox proportional hazards model for recovery to the remote preoperative baseline requiring at least 4 days of available Fitbit step count data per week

Predictor	Hazard Ratio (HR)	95% CI	P value
Age (per year)	0.96	0.94–0.99	0.01
BMI (per kg/m²)	0.99	0.96–1.02	0.52
Charlson Comorbidity Index, per point	0.96	0.89–1.05	0.37
Male sex (reference: female)	1.14	0.77–1.70	0.51
Surgery type: TKA (reference: THA)	0.96	0.62–1.49	0.86
Education: no college (reference: college)	1.24	0.58–2.64	0.58
Education: some college (reference: college)	1.2	0.76–1.88	0.43
Employment: working (reference: not working)	0.66	0.41–1.04	0.08
Income: low/skip (reference: high)	1.14	0.66–1.97	0.63
Income: mid (reference: high)	0.73	0.45–1.20	0.21
Marital status: not married (reference: married/living with partner)	0.86	0.56–1.32	0.49
Immediate preoperative activity level (per 1000 steps)	1.47	1.29–1.68	<0.001
Remote preoperative activity level (per 1000 steps)	0.68	0.59–0.78	<0.001

Table S9. Cox proportional hazards model for recovery to the immediate preoperative baseline requiring at least 4 days of available Fitbit step count data per week

Predictor	Hazard Ratio (HR)	95% CI	P value
Age (per year)	0.98	0.95–1.01	0.28
BMI (per kg/m²)	1	0.96–1.04	0.93
Charlson Comorbidity Index, per point	1	0.88–1.13	0.95
Male sex (reference: female)	0.75	0.46–1.22	0.24
Surgery type: TKA (reference: THA)	0.61	0.39–0.93	0.02
Education: no college (reference: college)	0.99	0.63–1.56	0.97
Education: some college (reference: college)	1.15	0.66–1.99	0.62
Employment: working (reference: not working)	0.88	0.55–1.40	0.58
Income: low/skip (reference: high)	1.68	0.98–2.87	0.06
Income: mid (reference: high)	1.46	0.90–2.38	0.13
Marital status: not married (reference: married/living with partner)	0.68	0.44–1.05	0.08
Immediate preoperative activity level (per 1000 steps)	0.91	0.81–1.02	0.09
Remote preoperative activity level (per 1000 steps)	1.06	0.96–1.18	0.26

Table S10. Bootstrap stability analysis of preoperative knots and phase-specific slopes.

Procedure	Selected knot, median (IQR)	Gradual decline phase slope, median (IQR)	Accelerated decline phase slope, median (IQR)
TKA	−55 (−57 to −53)	−6.5 (−11.6 to −2.7)	−20.7 (−23.4 to −18.2)
THA	−16 (−18 to −14)	−5.1 (−6.7 to −3.0)	−57.6 (−66.2 to −45.7)

Note. Values are medians with interquartile ranges from 200 participant-level bootstrap resampling iterations. Slopes represent weekly changes in daily step count during the corresponding phase. Knot locations are indexed in weeks relative to the arthroplasty date. TKA, total knee arthroplasty; THA, total hip arthroplasty; IQR, interquartile range.

Table S11. Bootstrap stability analysis of postoperative knots and phase-specific slopes.

Procedure	First knot, median (IQR)	Second knot, median (IQR)	Rapid recovery phase slope, median (IQR)	Decelerating recovery phase slope, median (IQR)	Plateau phase slope, median (IQR)
TKA	6 (5 to 7)	19 (19 to 21)	654.2 (598.6 to 738.5)	111.0 (95.6 to 128.9)	3.9 (1.9 to 5.5)
THA	6 (6 to 7)	19 (18 to 20)	909.4 (825.2 to 975.5)	83.6 (68.1 to 96.4)	−1.0 (−4.5 to 1.7)

Note. Values are medians with interquartile ranges from 200 participant-level bootstrap resampling iterations. Slopes represent weekly changes in daily step count during the corresponding phase. Knot locations are indexed in weeks relative to the arthroplasty date. TKA, total knee arthroplasty; THA, total hip arthroplasty; IQR, interquartile range.

Table S12. Cox proportional hazards model for recovery to the remote preoperative baseline, defined as 4 consecutive weeks at or above baseline

Predictor	Hazard Ratio (HR)	95% CI	P value
Age (per year)	0.96	0.92–0.99	0.01
BMI (per kg/m²)	0.97	0.92–1.03	0.32
Charlson Comorbidity Index, per point	0.9	0.78–1.03	0.13
Male sex (reference: female)	1.44	0.81–2.55	0.21
Surgery type: TKA (reference: THA)	0.77	0.43–1.35	0.36
Education: no college (reference: college)	1.4	0.52–3.76	0.5
Education: some college (reference: college)	1.24	0.65–2.35	0.51
Employment: working (reference: not working)	0.57	0.32–1.04	0.07
Income: low/skip (reference: high)	1.74	0.86–3.51	0.12
Income: mid (reference: high)	1.13	0.62–2.04	0.69
Marital status: not married (reference: married/living with partner)	1.0	0.58–1.74	>0.99
Immediate preoperative activity level (per 1000 steps)	1.54	1.31–1.81	<0.001
Remote preoperative activity level (per 1000 steps)	0.62	0.53–0.74	<0.001

Note. HR, hazard ratio; CI, confidence interval; TKA, total knee arthroplasty; THA, total hip arthroplasty; BMI, body mass index. Recovery to the remote preoperative baseline was defined as the first of 4 consecutive weeks in which weekly averaged daily step count reached or exceeded the mean daily step count during weeks −104 to −55 before surgery. HRs greater than 1 indicate a greater likelihood of achieving recovery. Immediate preoperative activity level was defined as the mean daily step count during weeks −4 to −1 before surgery.

Table S13. Cox proportional hazards model for recovery to the immediate preoperative baseline, defined as 4 consecutive weeks at or above baseline

Predictor	Hazard Ratio (HR)	95% CI	P value
Age (per year)	0.98	0.95–1.01	0.15
BMI (per kg/m²)	1	0.96–1.04	0.98
Charlson Comorbidity Index, per point	0.99	0.87–1.12	0.83
Male sex (reference: female)	0.75	0.46–1.21	0.24
Surgery type: TKA (reference: THA)	0.59	0.38–0.90	0.01
Education: no college (reference: college)	1.03	0.67–1.60	0.89
Education: some college (reference: college)	1.04	0.62–1.76	0.87
Employment: working (reference: not working)	0.87	0.55–1.38	0.56
Income: low/skip (reference: high)	1.52	0.87–2.63	0.14
Income: mid (reference: high)	1.41	0.87–2.29	0.17
Marital status: not married (reference: married/living with partner)	0.68	0.44–1.06	0.09
Immediate preoperative activity level (per 1000 steps)	0.9	0.80–1.01	0.07
Remote preoperative activity level (per 1000 steps)	1.08	0.97–1.20	0.18

Note. HR, hazard ratio; CI, confidence interval; TKA, total knee arthroplasty; THA, total hip arthroplasty; BMI, body mass index. Recovery to the immediate preoperative baseline was defined as the first of 4 consecutive weeks in which weekly averaged daily step count reached or exceeded the mean daily step count during weeks −4 to −1 before surgery. HRs greater than 1 indicate a greater likelihood of achieving recovery. Remote preoperative activity level was defined as the mean daily step count during weeks −104 to −55 before surgery.
